# A study of flavonoid inhibitors against Monkeypox H1 phosphatase

**DOI:** 10.1080/14756366.2025.2535585

**Published:** 2025-08-12

**Authors:** Hwa Young Kim, Mi-Sun Kim, Dong Hae Shin

**Affiliations:** College of Pharmacy and Graduates School of Pharmaceutical Sciences, Ewha W. University, Seoul, Republic of Korea

**Keywords:** Dual-specific phosphatase, monkeypox virus, flavonoids, docking analysis, phosphatase inhibitors

## Abstract

Poxviruses regulate their replication cycle through host phosphorylation pathways, with dual-specific phosphatase H1(DUSP-H1) playing a key role in immune evasion by dephosphorylating STAT1 and inhibiting interferon(IFN) responses. Given its high conservation across orthopoxviruses, it represents a promising antiviral target. This study screened a flavonoid library against DUSP-H1 from monkeypox virus (*m*DUSP-H1) using a malachite green-based phosphatase assay, identifying Myricetin, (−)-Gallocatechin, Cupressuflavone, (−)-Epigallocatechin gallate, Baicalein, and Herbacetin as potent *m*DUSP-H1 inhibitors (IC_50_: 7.07–14.05 μM). Docking analysis revealed key hydrogen bonding interactions between 5,7-hydroxyl groups of the hydroxyflavone backbone and Asp79 and Arg116 of *m*DUSP-H1, respectively. Additional interactions with Ser23 via the 3’-hydroxyl group seems to enhance binding and effectively blocking the enzyme’s active site. These findings align with previous studies on tyrosine phosphatase inhibitors, supporting flavonoids as broad-spectrum viral phosphatase inhibitors. Further structural and pharmacokinetic studies will aid in developing optimised antiviral therapies against monkeypox, variola, and cowpox viruses.

## Graphical Abstract

**Figure:**
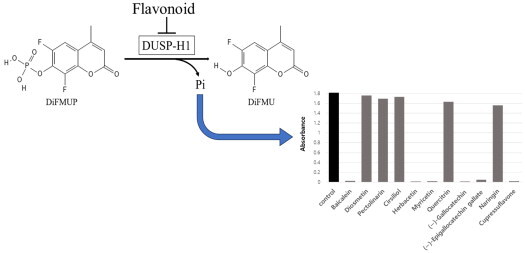

## Introduction

Monkeypox, first identified in the Democratic Republic of the Congo in 1970, is a zoonotic virus that continues to pose a significant threat to human health. The global outbreak in 2022 was highly unusual, given that past cases were primarily confined to endemic regions. This widespread outbreak suggests that continuous transmission between animals and humans, as well as among humans, is increasing, raising concerns that monkeypox could become a more persistent global threat, much like COVID-19. As of September 16, 2023, a total of 90,439 confirmed cases and 157 deaths had been reported from the 2022–2023 outbreak^1^.

In this study, we targeted dual-specific phosphatase (DUSP)-H1, a subset of protein tyrosine phosphatases that removes phosphate groups from phosphoserine/threonine and phosphotyrosine residues^2–4^. DUSP-H1 plays a key role in suppressing host immunity by dephosphorylating signal transducer and activator of transcription 1 (STAT1), thereby interfering with interferon. Interestingly, approximately 200 copies of DUSP-H1 are found in newly formed monkeypox viral particles, indicating its critical function during the early stages of infection^5^. Since DUSP-H1 expression strongly correlates with viral infectivity, it presents a promising target for developing anti-poxvirus therapeutics.

Recent studies have determined the X-ray crystal structure of monkeypox virus DUSP-H1 (*m*DUSP-H1) at 1.8 Å resolution^6^. The enzyme adopts a domain-swapped dimeric structure, exhibiting structural similarities to DUSP-H5 and -H27. The active site comprises an Asp79-Cys110-Arg116 catalytic triad. Notably, DUSP-H1 sequences are highly conserved among orthopoxviruses, including vaccinia virus, variola virus, and cowpox virus, suggesting that DUSP-H1 inhibitors may have broad-spectrum antiviral potential^7^.

**Figure 1.**
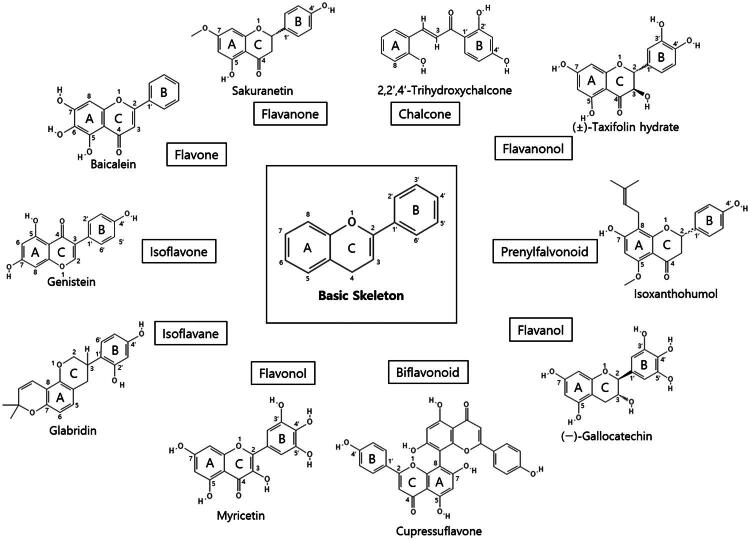
Representative skeletal structures of flavonoids. This picture illustrates ten distinct scaffolds using representative flavonoid derivatives. These structures highlight their fundamental frameworks along with the carbon atom numbering patterns.

In this study, we screened a series of flavonoids for their potential to inhibit *m*DUSP-H1 activity (Figure 1). Flavonoids are widely recognised for their diverse biological activities, including direct antiviral effects through the inhibition of viral enzymes^8^. We employed a malachite green-based phosphatase assay to assess *m*DUSP-H1 inhibition, adapting a workflow similar to that used in our previous inhibitor studies. Here, we report several flavonoid compounds that effectively inhibit *m*DUSP-H1 activity and propose a plausible mechanism of inhibition.

## Materials and methods

### Materials and reagents

Luria-Bertani (LB) medium components were obtained from Condalab (Madrid, Spain). Ampicillin, glycerol, and isopropyl-β-D-1-thiogalactopyranoside (IPTG) were purchased from Sigma (St. Louis, MO, USA). For protein purification, Tris-HCl, NaCl, imidazole, phenylmethylsulfonyl fluoride, and DNase I were obtained from Sigma (St. Louis, MO, USA). The ultrasonic cell disruptor (Digital Sonifier 450) was purchased from Branson (USA). The ÄKTA explorer system and His-Trap column were from GE Healthcare (Piscataway, NJ, USA). The malachite green reagent components, other assay reagents, and all 64 flavonoids used in both the malachite green assay and inhibitory assay were purchased from Sigma-Aldrich (St. Louis, MO, USA). The 0.22 μm PVDF syringe filter was obtained from Youngin Frontier (Daejeon, Korea). The Schrödinger software suite (Maestro, version 14.2) was licensed from Schrödinger, LLC (New York, NY, USA).

### Protein expression of DUSP-H1

The coding sequence of *m*DUSP-H1 (NCBI. https://www.ncbi.nlm.nih.gov/. Ref. Seq. QGQ59814.1) was chemically synthesised by Bioneer (Daejeon, Korea) and subsequently cloned into the pBT7-based expression vector Bioneer (Daejeon, Korea), which utilises a T7 promoter for high-level expression in *E. coli*. The resulting plasmid DNA was transformed into *E. coli* BL21 (DE3) cells for protein expression. *E. coli* BL21 (DE3) cells were purchased from Enzynomics (Daejeon, Korea). For initial bacterial growth, *E. coli* BL21 (DE3) cells were plated on Luria–Bertani (LB) agar supplemented with 150 μg/ml ampicillin. Several individual colonies were selected and inoculated into 10 ml LB broth containing 150 μg/ml ampicillin, followed by incubation under standard shaking conditions. To establish long-term stocks, 0.85 ml of each culture was mixed with 0.15 ml glycerol and stored at 193 K for future large-scale culture inoculation.

For large-scale expression, frozen cell stocks were revived in 5 ml LB medium, followed by dilution into 1000 ml fresh LB medium. The cultures were incubated at 310 K with shaking until they reached an OD_**600**_ of 0.6–0.8, indicating the appropriate phase for protein induction. At this point, isopropyl-β-d-1-thiogalactopyranoside (IPTG) was added to a final concentration of 1 mM to induce *m*DUSP-H1 expression. The cultures were further incubated at 289 K for 16 h in a shaking incubator to allow optimal protein expression.

Following induction, cells were harvested by centrifugation at 7,650 g (6,500 rpm) for 10 min in a high-speed refrigerated centrifuge at 277 K, ensuring efficient cell pellet collection for subsequent protein purification steps.

### Protein purification of DUSP-H1

The harvested cell paste was resuspended in 30 ml of lysis buffer containing 50 mM Tris–HCl (pH 8.0), 100 mM NaCl, 10 mM imidazole, 1 mM phenylmethylsulfonyl fluoride, and 10 μg/ml DNase I. The cell suspension was then disrupted using an ultrasonic cell disruptor (Digital Sonifier 450, Branson, USA) to ensure efficient cell lysis. Following sonication, the cell debris was removed by centrifugation at 24,900 g (15,000 rpm) for 30 min using a high-speed refrigerated ultracentrifuge at 277 K. The resulting supernatant, containing the soluble fraction of the expressed *m*DUSP-H1, was subjected to affinity purification.

Purification was performed using an ÄKTA explorer system (GE Healthcare, Piscataway, NJ) equipped with a His-Trap column (GE Healthcare, Piscataway, NJ), allowing for efficient His-tag affinity chromatography and recovery of purified *m*DUSP-H1.

### Preparation of malachite green reagent

The malachite green reagent was prepared by mixing 4.2% (w/v) ammonium molybdate in 4 M HCl, 0.045% (w/v) malachite green, and 1% (v/v) Tween 20 in a 1:3:0.1 ratio. These components were combined and stirred for a minimum of 30 min to ensure complete dissolution. Following thorough mixing, the solution was syringe-filtered through a 0.22 μm PVDF syringe filter (Youngin Frontier) to remove any particulates, ensuring clarity and consistency.

Initially, the reagent appears dark brown, but upon standing at room temperature for 1 h, it transitions to a golden yellow colour, indicating that it is fully stabilised and ready for use. This colour change serves as a visual confirmation of the reagent’s readiness, ensuring its proper functionality in subsequent applications.

### Chemical screening with a malachite green assay

A malachite green assay was utilised to screen flavonoid compounds for their potential effects on *m*DUSP-H1 activity. The assay is based on the enzymatic hydrolysis of 6,8-difluoro-7-hydroxy-4-methylcoumarin phosphate (DiFMUP) by *m*DUSP-H1, which results in the release of phosphate and the formation of 6,8-difluoro-7-hydroxy-4-methylcoumarin (DiFMU). The released phosphate was subsequently detected using the malachite green method.

In the experimental procedure, 160 μl of malachite green solution was added to 40 μl of the *m*DUSP-H1-catalyzed reaction mixture. The mixture was then incubated at room temperature for 10 min to allow the development of the characteristic colour change. Following incubation, the absorbance was measured at 620 nm using a spectrophotometer. The amount of phosphate released was quantified by comparison with a standard curve generated using known concentrations of inorganic phosphate.

This method enabled a quantitative assessment of *m*DUSP-H1 activity, allowing for the identification of potential inhibitors or modulators among the screened chemical compounds. The malachite green assay provided a reliable and sensitive means of detecting phosphate release, making it a valuable tool for evaluating enzyme kinetics and compound interactions in the study of *m*DUSP-H1 function.

### Inhibitor search against mDUSP-H1

Each compound was tested at multiple concentrations within its soluble range in the enzyme mixture. The assay mixture consisted of 50 mM Tris (pH 7.5), 10 mM MgCl_2_, 0.01% Triton X-100, 0.025 mg/ml *m*DUSP-H1, and distilled water. For the assay, 40 μl of each compound concentration was added to the wells of a test plate and incubated at room temperature for 1 h to allow interaction with *m*DUSP-H1. Following this incubation, 0.25 mM DiFMUP, the substrate of *m*DUSP-H1, was introduced into the wells under identical conditions and left to react for an additional 1 h at room temperature. This experimental setup facilitated the evaluation of inhibitory potential by comparing enzyme activity levels across different concentrations of each candidate compound.

### Ligand preparation, target preparation, and docking strategy

All docking and scoring calculations were conducted using the Schrödinger software suite (Maestro, version 14.2). The test compounds were obtained from the PubChem (https://pubchem.ncbi.nlm.nih.gov/) database in SDF format and consolidated into a single file. This file was then imported into Maestro and prepared using LigPrep, ensuring the generation of valid, low-energy 3D molecular structures. The atomic coordinates of the *m*DUSP-H1 crystal structure (PDB ID: 8GZ4) were retrieved from the Protein Data Bank (https://www.rcsb.org/). Since the crystal structure of *m*DUSP-H1 was determined in an apo form, the side-chain sulfur atom of the catalytic cysteine residue (Cys110) was selected as the centre of the docking grid, based on its role within the conserved catalytic triad consisting of Asp79, Cys110, and Arg116. The structure was preprocessed using the Protein Preparation Wizard, including the removal of solvent molecules, addition of hydrogen atoms, and restrained energy minimisation. Protonation and ionisation states of the ligands were assigned at physiological pH (7.0 ± 2.0) using Epik designed to predict the protonation and ionisation states of drug-like molecules at a given pH. The receptor grid was defined with an inner box of 10 Å to accommodate ligand centroids and an outer box of 30 Å to encompass the entire ligand. Initial docking was carried out using Glide in standard precision (SP) mode to broadly sample potential ligand binding orientations, followed by refinement of the top-ranked poses with Glide extra precision (XP) mode to improve scoring accuracy and pose discrimination^8^.

Induced-Fit Docking (IFD) was further employed to account for receptor flexibility and optimise the modelling of ligand–protein interactions. Following the method described by Sherman et al. (2006)^9^, the prepared ligand conformers were initially docked using Glide SP mode to generate candidate poses. Protein side chains and backbone residues within 5.0 Å of each ligand pose were then refined using Prime^10^, allowing for conformational adjustments to better accommodate the ligand. A set of induced-fit receptor conformations within 30.0 kcal/mol of the lowest-energy structure was retained, and the ligands were subsequently redocked into these refined receptor states. Finally, Prime MM-GBSA (Molecular Mechanics–Generalized Born Surface Area)^11^ and Glide Score were applied to evaluate the binding affinities and interaction stability of the final poses. This combined docking protocol ensured high reliability in predicting ligand–protein binding interactions.

## Results/

### Protein expression and purification of DUSP-H1

The yield of cells harvested for the purification of *m*DUSP-H1 was 2.19 g per 1000 ml of *E. coli* culture. The protein was purified through affinity chromatography using a 5 ml His-Trap column (GE Healthcare, Piscataway, New Jersey, USA). Prior to purification, the column was equilibrated with a buffer containing 50 mM Tris–HCl (pH 8.0), 300 mM NaCl, and 10 mM imidazole. The target protein was then eluted using a gradient of 10 to 500 mM imidazole in a buffer composed of 50 mM Tris–HCl (pH 8.0) and 100 mM NaCl.

Following elution, the purified protein was buffer-exchanged into 20 mM Bis-Tris (pH 7.0) using a Vivaspin 20 MWCO 10 kDa centrifugal device (GE Healthcare) to ensure optimal conditions for downstream applications. SDS–PAGE analysis confirmed successful purification, showing a single band at approximately 22 kDa, corresponding to the expected molecular weight of *m*DUSP-H1. The protein was subsequently concentrated to 27.3 mg/mL in 20 mM Bis-Tris (pH 7.0) for use in the malachite green assay.

### Chemical screening with a malachite green assay

A flavonoid library comprising ten different scaffolds was constructed (Figure 1). This library included five isoflavones, one isoflavane, eighteen flavones, eight flavonols, seven flavanols, seven flavanones, two flavanonols, one prenylflavonoid, two flavonolignans, six chalcones, three biflavonoidand, and four unclassified flavonoids (Table S1). The library was applied to assess the inhibitory activity of flavonoids against *m*DUSP-H1. A total of sixty-four flavonoids were screened, each at a concentration of 40 μM, to evaluate their inhibitory effects. Since flavonoids are known to form aggregates through complexation, resulting in non-specific inhibition of various enzymes, Triton X-100 was consistently included in the assay to ensure the specificity of inhibition^12^. It is known that 0.01% Triton X-100 does not affect phosphatase activity^13^.

**Figure 2.**
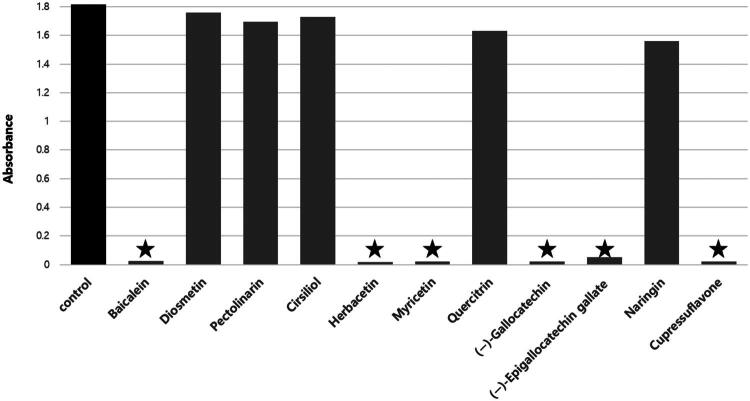
Comparison of *m*DUSP-H1 reactivity with various flavonoids. The reactivity of *m*DUSP-H1 with various flavonoids was assessed using the malachite green assay with 0.25 mM DifMUP as a substrate. All flavonoids were used at 25 μM concentration. Absorbance measurements were used to evaluate *m*DUSP-H1 activity against flavonoids, as shown in the graph. Compared to the DMSO control, *m*DUSP-H1 exhibited significantly reduced reactivity in the presence of baicalein, herbacetin, myricetin, (−)-gallocatechin, (−)-epigallocatechin gallate, and cupressuflavone. In contrast, its reactivity remained relatively unchanged with diosmetin, pectolinarin, cirsiliol, quercitrin, and naringin. ★ Indicates significant inhibition (*p* < 0.05) compared to the control.

Several flavonoids exhibiting prominent inhibitory activity were selected for further analysis (Figure 2). The inhibitory effects of candidate compounds on *m*DUSP-H1 activity were assessed by plotting their activity against varying inhibitor concentrations.

### IC_50_ values of candidate flavonoids

**Figure 3.**
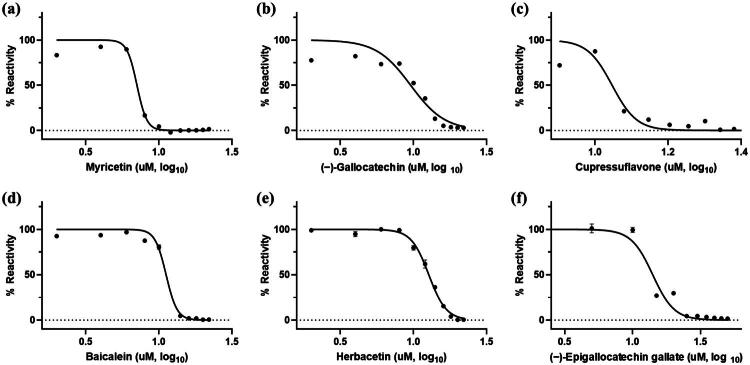
Dose-dependent inhibitory curves of potent inhibitors. (a) Myricetin, (b) (−)-gallocatechin, (c) cupressuflavone, (d) baicalein, (e) herbacetin and (f) (−)-epigallocatechin gallate. Each data point represents the dose-dependent response against *m*DUSP-H1, with values expressed as the mean ± standard error of the mean (*n* = 3).

Among the tested compounds, Myricetin, (−)-Gallocatechin, Cupressuflavone, Baicalein, Herbacetin, and (−)-Epigallocatechin gallate demonstrated outstanding inhibitory activity against *m*DUSP-H1. The binding affinity data were plotted as log inhibitor concentration *versus* percent fluorescence inhibition (Figure 3), revealing that these flavonoids significantly reduced fluorescence intensity, thereby confirming their *m*DUSP-H1 inhibitory activity. The IC_50_ values, determined from dose-dependent inhibitory curves, were as follows: Myricetin (7.08 μM), (−)-Gallocatechin (9.56 μM), Cupressuflavone (11.13 μM), Baicalein (11.19 μM), Herbacetin (12.71 μM), and (−)-Epigallocatechin gallate (14.05 μM).

### Docking results and induced-Fit evaluation of flavonoid inhibitors against mDUSP-H1

Docking analysis was conducted to evaluate the binding affinity and pose prediction of six natural compounds, whose IC_50_ values had been determined against *m*DUSP-H1. Using Glide standard precision (SP) docking to broadly sample ligand conformations, followed by extra precision (XP) docking to refine the highest-ranked poses, Myricetin (−6.0 kcal/mol) showed the most favourable XP docking scores, suggesting strong potential interactions with the catalytic triad centred on Cys110. Cupressuflavone exhibited less favourable XP score, indicating weaker predicted binding (Table 1).

**Table 1.** IC_50_ values, docking scores (rigid and induced fit), and chemical structures of *m*DUSP-H1 inhibitory compounds.

No.	Compound	IC_50_ (μM)	Docking score	IFD Score	Structure
1	Myricetin	7.08 ± 0.09	−6.069	−7.367	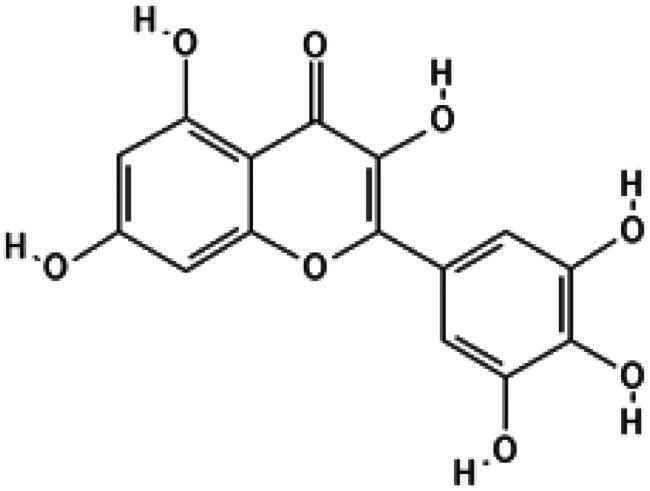
2	(−)-Gallocatechin	9.56 ± 0.32	−4.23	−6.602	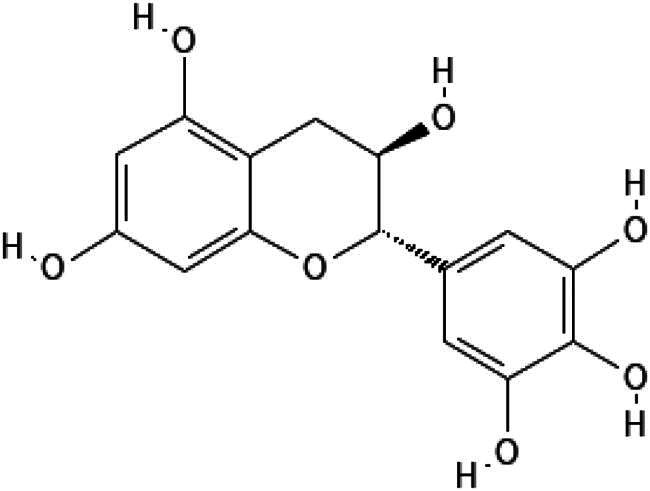
3	Cupressuflavone	11.13 ± 0.18	−1.44	−6.092	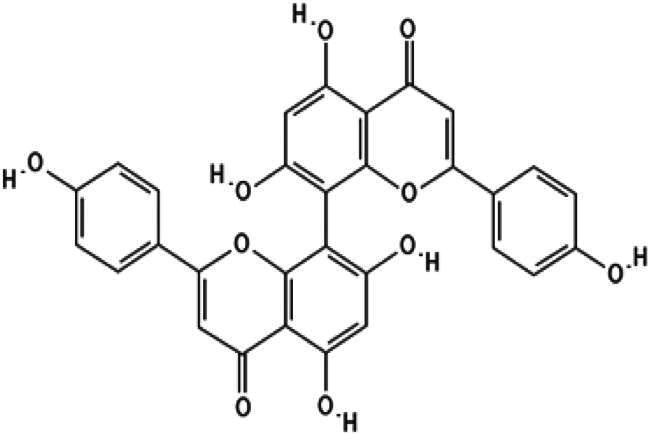
4	Baicalein	11.19 ± 0.14	−4.578	−5.766	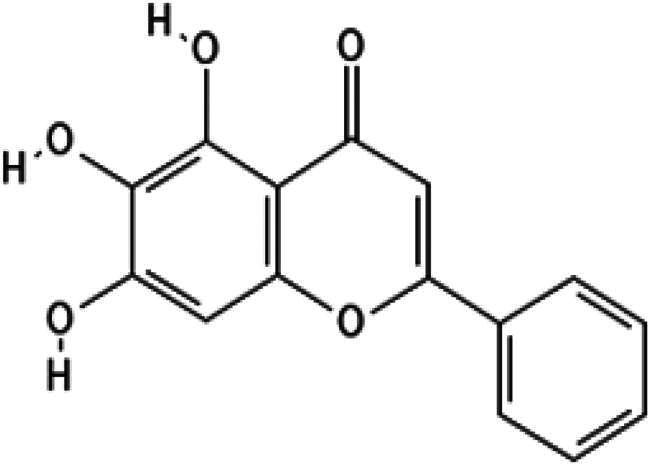
5	Herbacetin	12.71 ± 0.08	−3.937	−5.618	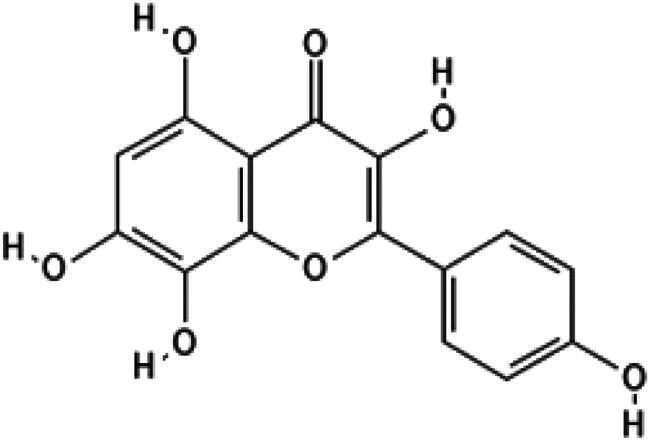
6	(−)-Epigallocatechin gallate	14.05 ± 0.35	−4.888	−5.148	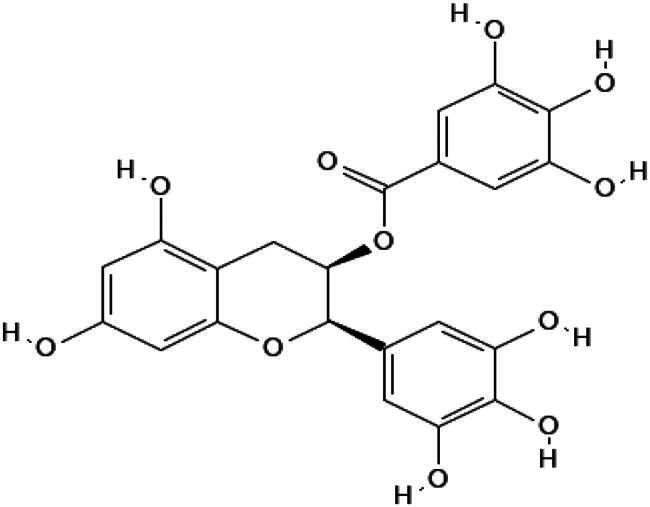

To account for receptor flexibility, IFD was performed, beginning with SP docking to generate initial poses, followed by Prime-based side-chain and backbone refinement within 5.0 Å of each ligand, and redocking into receptor conformations within 30.0 kcal/mol of the global minimum. The IFD workflow produced a similar rank ordering to the XP results but revealed enhanced hydrogen bonding and improved steric accommodation for Myricetin and (−)-Gallocatechin, as evidenced by more favourable Prime MM-GBSA binding energies and final IFD docking scores. The IFD-derived scores demonstrated a strong correlation with the experimentally determined IC_50_ values.

## Discussion

Poxviruses use mimicry of host biology of phosphorylation and de-phosphorylation for the regulation of their viral life cycle. The Variola virus phosphatase H1 plays a critical role in viral replication by dephosphorylating the transcription factor Stat1 and thereby blocks interferon (IFN)-stimulated innate immune responses^14^. Given the high conservation of DUSP-H1 across orthopoxviruses, including the monkeypox virus, it represents a promising antiviral target for the development of broad-spectrum inhibitors. In addition, since their functional role is quite important in the pathogenicity of Poxvirus, they are good candidates for developing anti-Poxvirus agents.

In this study, *m*DUSP-H1 was screened using a flavonoid library to explore potential inhibitory interactions. Although flavonoids are extensively studied for their anticancer and antioxidant activities, their clinical development remains challenging due to issues such as cytotoxicity and limited bioavailability^15^^,^^16^. Despite their ability to selectively induce cytotoxicity in cancer cells, high concentrations may also damage normal cells by promoting oxidative stress and mitochondrial dysfunction. This dual nature complicates the establishment of safe and effective dosing regimens, thereby limiting their therapeutic potential. Nevertheless, the ability of flavonoids to bind specific enzymes, supported by available crystal structures, offers a promising foundation for the rational design of novel therapeutics targeting disease-associated enzymes.

The selection of the 64 flavonoids in this study was guided by a rationale aimed at maximising structural diversity, pharmacological relevance, and screening efficiency. The library spans major flavonoid subclasses, including isoflavones, isoflavane, flavones, flavonols, flavanols, flavanones, flavanonols, chalcones, biflavonoids and rarer types such as prenylflavonoid, flavonolignans and unclassified ones. This broad coverage enables comprehensive structure–activity relationship (SAR) analysis across chemically distinct scaffolds. Selection favoured compounds with known or predicted bioactivity such as anticancer, antioxidant, and enzyme-inhibitory properties based on existing literature and natural abundance. Therefore, the library was designed for identifying lead compounds suitable for a screening platform. Interestingly, six flavonoids displaying inhibitory activity of phosphatase activity of *m*DUSP-H1 and two flavonoids turned out to be most effective. (Table 1) Myricetin and (-)-gallocatechin clearly represent a function to suppress the activity of mDUSP-H1. Their IC_50_ values are 7.08 μM to 9.56 μM, respectively. Given their low cytotoxicity in normal human cell lines, with IC_50_ values generally exceeding 100 μM^17^^,^^18^, these compounds remain well below established cytotoxic thresholds, indicating a potentially favourable therapeutic window for further development. Myricetin has shown IC_50_ values greater than 100 μM in normal cell types such as HEK293 and human dermal fibroblasts (HDF), while retaining selective cytotoxic effects against certain cancer cells^19^. Similarly, (−)-Gallocatechin is considered non-toxic up to concentrations of 100 μM in normal human cells and is widely consumed through dietary sources like green tea^20^.

**Figure 4.**
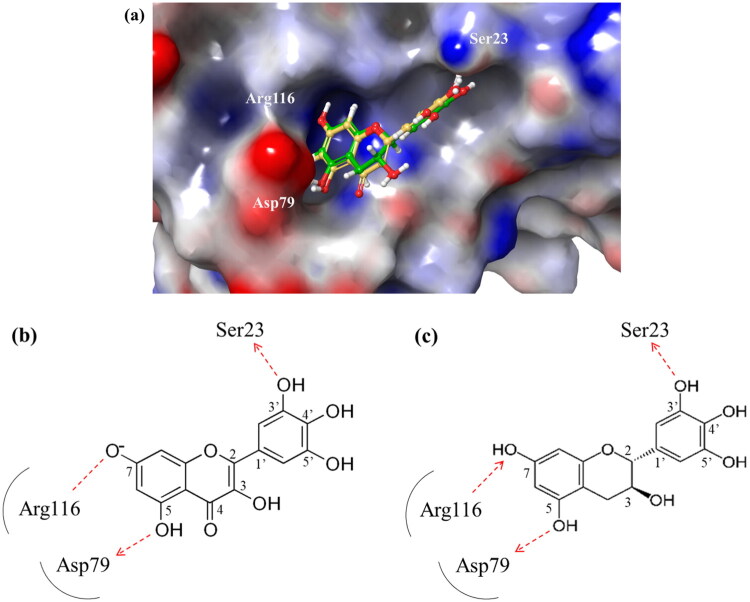
Docking-based binding mode of Myricetin and (−)-Gallocatechin with *m*DUSP-H1. (a) The docking poses depict myricetin (yellow) and (−)-gallocatechin (green) on the electrostatic surface potential of *m*DUSP-H1, where red indicates negative regions, blue represents positive regions, and white denotes uncharged areas. Additionally, 2D schematic representations of the docked flavonoid compounds with *m*DUSP-H1 are provided. (b) Myricetin and (c) (−)-gallocatechin interactions with key side chains of *m*DUSP-H1 are shown, with carbon atom labels referenced in the text. Red arrows indicate hydrogen bonds.

The core scaffold presumed to inhibit catalytic activity of *m*DUSP-H1 can be deduced from the two flavonoids. They possess three hydroxyl groups at the 3, 5, 7-positions of the basic flavonoid ring. They also have the same 3′, 4′, 5′-trihydroxy phenyl moiety. This core architecture seems essential for their inhibitory activity. The docking results from Myricetin and (−)-Gallocatechin were illustrated in Figure 4. The key interactions were identified between the 5-hydroxyl and 7-hydroxyl groups of the 3,5,7-hydroxyflavone backbone. These groups form hydrogen bonds with Asp79 and Arg116, respectively, anchoring the flavonoids at the active site entrance. This strategic positioning overlaps with the substrate-binding region, effectively blocking access to the catalytic pocket. These findings suggest that these flavonoids exert their inhibitory effect on *m*DUSP-H1 through competitive binding.

Furthermore, docking models reveal that the 3′-hydroxyl group of the trihydroxyphenyl moiety enhances binding affinity by forming an additional hydrogen bond with Ser23, a key residue within the substrate-binding site. Given that the active site of *m*DUSP-H1 is located on the enzyme surface and features a shallow cleft, this additional hydrogen bond likely contributes to the stability of the flavonoid-enzyme interaction. In contrast, the 4′-hydroxyl and 5′-hydroxyl groups of the hydroxyphenyl moiety remain solvent-exposed, suggesting a minimal role in binding interactions. The absence of the 3′-hydroxyl group may explain the slightly lower binding affinities observed for Baicalein (IC_50_ = 11.19 μM) and Herbacetin (IC_50_ = 12.71 μM). The docking poses of these molecules indicate that, although their overall binding modes are similar to those of Myricetin and (−)-Gallocatechin, their hydroxyphenyl moieties are oriented towards the solvent-accessible area due to the absence of hydrogen bonding with Ser23. In the cases of Cupressuflavone (IC_50_ = 11.13 μM) and (−)-Epigallocatechin gallate (IC_50_ = 14.05 μM), their additional bulky substituents appear to influence binding interactions. Docking models reveal notably different poses for these two compounds compared to the other four flavonoids. For (−)-Epigallocatechin gallate, the 5,7-dihydroxy groups of the chromen moiety form hydrogen bonds with Asp79 and Arg116, but no interaction with Ser23 is observed, likely due to structural constraints. The binding pose of Cupressuflavone is distinctly different from the others, primarily due to steric hindrance caused by its bulky additional groups. Notably, the 3-hydroxyl group of flavonol and flavanol, a key structural motif, does not appear to be essential for interactions with *m*DUSP-H1, as binding is also observed with non-flavonol and flavanol compounds. Despite these structural variations, the docking study strongly suggests that the presence of 5,7-hydroxyl groups in the hydroxyflavone backbone is a key structural determinant for inhibiting the catalytic activity of *m*DUSP-H1.

The inactivity of certain compounds can be partially explained by comparing their binding interactions with those of the active compounds identified in this study. Diosmetin, a flavone with a 5,7-dihydroxyflavone core and a 4′-methoxy group replacing the 3′,4′,5′-trihydroxyphenyl moiety present in Myricetin, showed no significant inhibitory activity, underscoring the importance of the 3′-hydroxyl group in stabilising key interactions, particularly with Ser23. Likewise, Quercitrin, which retains the aglycone structure of Quercetin but carries a rhamnose sugar at the 3-position, exhibited minimal inhibition. This suggests that bulky substitutions at this position may hinder optimal binding through steric effects or by disrupting critical hydrogen bonding within the catalytic cleft. Naringin, a flavanone glycoside, also demonstrated little to no activity, reinforcing the necessity of both a planar flavone or flavonol scaffold and a specific hydroxylation pattern—especially the 3′,4′,5′-trihydroxy configuration on the B-ring—for effective binding and inhibition. Together, these observations indicate that glycosidic modifications and alterations to the hydroxylation pattern can markedly reduce binding affinity and enzymatic inhibition.

Previous studies on tyrosine phosphatase inhibitors have demonstrated that polyphenolic compounds, including flavonoids, can effectively target viral phosphatases. Our findings align with these observations and support the need for broader evaluation of flavonoids across additional viral phosphatase targets. Notably, the viral DUSP-H1 adopts a domain-swapped dimer configuration and possesses unique structural features, such as surface-exposed catalytic residues and a shallow binding cleft. These characteristics distinguish it from human DUSPs like DUSP1 (MKP-1)^21^ and DUSP6^22^. As a result, structural comparison analyses suggest that the off-target effects of flavonoids on human DUSPs can be minimised, offering a promising path towards the development of selective inhibitors that specifically target viral DUSP-H1.

Docking results indicate a potential key structural feature of flavonoid inhibitors against *m*DUSP-H1, providing a promising direction for further investigation. Additionally, assessing the pharmacokinetic properties of these flavonoids in *in vivo* models will be essential to evaluate their bioavailability, metabolic stability, and therapeutic potential, ultimately guiding the development of optimised anti-poxvirus drugs. The crystallographic study of *m*DUSP-H1 complexed with the above six flavonoids is going on to corroborate their binding poses and to use as a template for developing better inhibitory compounds.

## Conclusion

This study highlights flavonoids as promising inhibitors of *m*DUSP-H1, a conserved phosphatase essential for monkeypox virus immune evasion. Compounds such as Myricetin and (−)-Gallocatechin demonstrated strong inhibitory activity, corroborated by docking analyses that revealed key interactions with catalytic residues. Our work centres on the biochemical characterisation of *m*DUSP-H1 and the identification of its inhibitors through an *in vitro* enzymatic assay, representing a crucial initial step in target validation and compound screening. These findings offer valuable structural insights for the rational design of selective inhibitors and support the development of broad-spectrum antivirals against orthopoxvirus infections.

## Supplementary Material

Supplementary_table_S1.docx

## Data Availability

The authors confirm that the data supporting the findings of this study are available within the article and its supplemental materials.
