# Supplementary material for: A study of flavonoid inhibitors against Monkeypox H1 phosphatase
Source: J Enzyme Inhib Med Chem. 2025 Aug 12;40(1):2535585. doi: 10.1080/14756366.2025.2535585 (PMC12344671; doi:10.1080/14756366.2025.2535585)
Supplement: Supplementary_table_S1.docx [file IENZ_A_2535585_SM7407.docx]

|  | No | Name of compound |  | No | Name of compound |
| --- | --- | --- | --- | --- | --- |
| Isoflavone | 1 | Daidzein | Prenylflavonoid | 33 | Isoxanthohumol |
|  | 2 | Genistein | Flavanol | 34 | (−)-Gallocatechin |
|  | 3 | Genistin |  | 35 | (±)-Epigallocatechin gallate |
|  | 4 | Ipriflavone |  | 36 | (−)-Epicatechin |
|  | 5 | Puerarin |  | 37 | (±)-Catechin |
| Isoflavane | 6 | Glabridin |  | 38 | (−)-Gallocatechin gallate |
| Flavone | 7 | Baicalein |  | 39 | (−) Catechin gallate |
|  | 8 | Diosmin |  | 40 | (+)-Catechin hydrate |
|  | 9 | Diosmetin | Flavanone | 41 | Hesperidin |
|  | 10 | Skullcapflavone II |  | 42 | Naringenin |
|  | 11 | beta-Naphthoflavone |  | 43 | Sakuranetin |
|  | 12 | Orientin |  | 44 | Naringin |
|  | 13 | Acacetin |  | 45 | Poncirin |
|  | 14 | Baicalin |  | 46 | Bavachin |
|  | 15 | Rhoifolin |  | 47 | Flavanone |
|  | 16 | Hispidulin | Flavanonol | 48 | (±)-Taxifolin hydrate |
|  | 17 | Sinensetin |  | 49 | Astilbin |
|  | 18 | Oroxin B | Flavonolignan | 50 | Silymarin |
|  | 19 | Pectolinarin |  | 51 | Silibinin |
|  | 20 | Cirsiliol | Chalcone | 52 | Isobavachalcone |
|  | 21 | Homoplantaginin |  | 53 | 2,2′,4′-Trihydroxychalcone |
|  | 22 | Amentoflavone |  | 54 | Sofalcone |
|  | 23 | Luteolin |  | 55 | Neohesperidin dihydrochalcone |
|  | 24 | Apigenin |  | 56 | Helichrysetin |
| Flavonol | 25 | Herbacetin |  | 57 | Cardamonin |
|  | 26 | Kaempferol | Unclassified | 58 | Rhodamine 6G |
|  | 27 | Morin |  | 59 | Dienestrol |
|  | 28 | Myricetin |  | 60 | Mangiferin |
|  | 29 | Fisetin |  | 61 | Auraptene |
|  | 30 | Quercitrin | Biflavonoid | 62 | Cupressuflavone |
|  | 31 | Quercetin |  | 63 | Ginkgetin |
|  | 32 | Rutin |  | 64 | Hinokiflavone |

**Table S1.** A Flavonoid Library
